# Aquaporin 1 (AQP1) Expression in Healthy Dog Tears

**DOI:** 10.3390/ani10050820

**Published:** 2020-05-09

**Authors:** Barbara Lamagna, Paolo Ciaramella, Francesco Lamagna, Antonio Di Loria, Arturo Brunetti, Alessandra Pelagalli

**Affiliations:** 1 Department of Veterinary Medicine and Animal Production, University of Naples Federico II, 80137 Naples, Italy; blamagna@unina.it (B.L.); paociara@unina.it (P.C.); lamagna@unina.it (F.L.); antonio.diloria@unina.it (A.D.L.); 2 Department of Advanced Biomedical Sciences, University of Naples Federico II, 80131 Naples, Italy; brunetti@unina.it; 3 Institute of Biostructures and Bioimaging (IBB), National Research Council (CNR), 80131 Naples, Italy

**Keywords:** aquaporin (AQP), eye, tear fluid, dog, physiology

## Abstract

**Simple Summary:**

The characterisation of tear proteins is very important for scientists and clinicians, as it enhances their understanding of ocular physiological phenomena that sometimes evolve into diseases. Recently, ophthalmic research has been focused on aquaporins (AQPs), a family of water channel proteins that are largely ubiquitous in body tissues and are known for their role in water and small solute transport across cell membranes. Based on AQPs’ presumable role in the eye, the aim of the present study was to investigate the expression of aquaporin-1 (AQP1) by Western blot analysis in canine eye tears. To this end, we collected tears from both eyes of 15 healthy dogs by employing two tear collection methods: Schirmer tear strips (STS) and ophthalmic sponges (OS). Moreover, ocular parameters such as Schirmer tear test 1 (STT 1), intraocular pressure (IOP), and tear film break up time (BUT) were measured, and fluorescein and lissamine green staining were performed to uncover possible correlations among the aforementioned parameters. Our results showed that the expression of AQP1 in tears collected by both methods and expressed as multiple bands (measured by densitometry) was higher for the tears collected by OS than for those collected by STS. This work forms the basis of future studies aiming to understand and establish the involvement of AQPs in the production and secretion of tears.

**Abstract:**

Aquaporins (AQPs) are a family of thirteen membrane proteins that play an essential role in the transport of fluids across the cell plasma membrane. Recently, the expression of AQPs in different ocular tissues and their involvement in the pathophysiology of eye diseases, have garnered attention. Considering that literature on AQP expression in the lacrimal glands and their secretion is scarce, we aimed to characterise AQP1 expression in the tears of healthy dogs using two tear collection methods (Schirmer tear strips (STS) and ophthalmic sponges (OS)). Fifteen healthy dogs, free of ophthalmic diseases, were included in the study. Tear collection was performed by using STS in one eye and OS in the other. After the extraction of proteins from the tears, the expression of AQP1 was analysed by Western blotting. AQP1 was expressed as a band of 28 kDa. In addition, differences were observed in the expression of AQP1 and in the correlation between tear volume and protein concentration, in tears collected by the two different methods. Our results suggest that AQP1 has a specific role in tear secretion; further research is required to assess its particular role in the function of the ocular surface in eye physiology and pathology.

## 1. Introduction

In the past twenty years, the lacrimal gland of humans and other animal species has been studied extensively in terms of its role in tear fluid composition and secretion [1], in the context of either ocular physiology [2] or pathology (especially dry eye syndrome) [3]. The lacrimal gland produces a tear film of a complex mixture, whose components (mucous, aqueous and lipid layers) exhibit a highly variable composition with a predominant aqueous fraction secreted by acinar cells, which play a major role in the high tear fluid volume. Moreover, it has been shown that the particular composition of the tear fluid plays a critical role in ocular surface health [4]. In fact, the fluid expelled out of the acinar lumen can be affected by the transport of electrolytes, water, and proteins by ductal cells that lead to the production of an almost isotonic tear fluid, which is enhanced in terms of its potassium and chloride contents [5]. Tear fluid is affected in its dynamic by three key processes: Tear flow, evaporation and blinking. In addition, several factors, such as environmental conditions, hormonal control, autonomic nervous system activation, and the movement of water and solutes can influence its composition modifying its volume, specific chemical-physical characteristics and thus, influencing its numerous biological activities [6]. Recently, several hypotheses have been proposed to examine the role of aquaporins (AQPs), localised to the lacrimal gland, on the regulation of fluid trafficking, albeit their specific action mechanism has not been fully characterised. AQPs are a family of thirteen water channel proteins well characterised in terms of their physiological role in the transport of fluids to several tissues of different organisms [7]. The first AQP isolated in the eye was aquaporin-0 (AQP0) [8]. Its characterisation in lens fibre cells has helped to elucidate its dual functions: Role as a channel protein [9] and a cell–cell adhesion molecule [10,11]. These findings suggest the involvement of AQPs in the eye function. Immunolocalisation studies on the eye of different laboratory animals have demonstrated the presence of AQPs in several cellular types and elucidated their potential roles in: i) Maintaining water balance in ocular tissues, ii) regulating tear film osmolarity [12], iii) generating aqueous humour [13] and iv) controlling retinal homeostasis [12,14]. In particular, studies on the lacrimal gland have showed the presence of AQP5 in the apical membrane of acinar and duct cells and AQP4 in the contralateral basolateral membrane [15,16,17]. AQP1 mRNA is expressed in the cornea, lacrimal gland and Meibomian glands in rat [18]. Among different isolated AQP isoforms, AQP1 is involved in the corneal water transport across the endothelial barrier, via the active extrusion of fluid from the corneal stroma across the corneal endothelium; corneal thickness was considerably reduced in AQP1-null mice [19]. Recently, the critical role of AQP1 in aqueous humour production has been confirmed. Furthermore, it has been demonstrated that *Aqp1* disruption using the CRISPR-Cas9 system can reduce intraocular pressure, thus providing a novel treatment strategy for glaucoma [20]. Finally, AQP1 suppression down-regulates the biomechanical strength of sclera [21].

Contrary to laboratory animal models, the characterisation of the functional aspects of lacrimal glands and the possible functional role played by AQPs have been scarcely investigated in dogs. In particular, Broadwater et al. [22] explored the lacrimal gland physiology in relation to tear production in juvenile dogs, thereby demonstrating that aging can affect tear production. In addition, Karasawa et al. [23] demonstrated a wide-spread distribution of AQPs (AQP0, AQP1, AQP3–5 and AQP9) in different dog eye tissues, excluding those found in the lacrimal glands.

To our knowledge, the characterisation of these water channel proteins at the lacrimal glands level could be useful in the context of studying tear fluid abnormalities in dogs [24] and other domestic animals (e.g., horses and cats) [25,26]. Finally, a high similarity in amino acid sequence between dog AQP1 (isolated from the kidney and erythroblasts) and human AQP1 (91–94% identity) [27], offers multiple opportunities for translational studies contributing to an understanding of AQP involvement in tear fluid dynamics. The results of such studies would be important, as they would pave way to diagnose tear film abnormalities (Schirmer tear test (STT)) [28].

The aim of the present study was to investigate the presence of AQP1 in healthy dog tears by Western blotting. This technique is a viable alternative to the more widely used enzyme-linked immunosorbent assay (ELISA), as it is a reliable and easy methodology with a high specificity and low cost. The evaluation of the AQP1 expression was performed on tear samples collected using Schirmer tear strips (STS) or ophthalmic sponges (OS) in order to verify possible differences between the two methods. Moreover, ocular parameters (Schirmer tear test values, intraocular pressure, and tear film break up time), were also analysed to evaluate possible correlations between them and AQP1 expression.

## 2. Materials and Methods

### 2.1. Animals

Fifteen healthy dogs that were presented for a routine check-up and vaccination and subjected to physical and ophthalmic examinations at the Veterinary Teaching Hospital of the University of Naples Federico II (Italy) between October 2018 and December 2018, were included in this study. Their owners gave their consent for them to be included in the research and signed an agreement. The characteristics of the dogs (breed, age, weight and sex) included in the study are presented in Table 1. The inclusion criteria were: Normal slit lamp examination, tear film break up time (BUT) of >10 s, Schirmer tear test value (STT-1) of >12 mm in 1 min, and IOP value of <25 mm Hg. The exclusion criteria were: Presence of autoimmune diseases and recent ocular surgery.

In order to evaluate the health status of the dogs, a complete clinical examination and haematobiochemical screening were performed on each animal enrolled. The ambient temperature and humidity values were also recorded.

All studies conformed to the standards and procedures for the proper care and use of animals and the experimental protocol was approved by the Ethical Animal Care and Use Committee of the University of Naples Federico II, Department of Veterinary Medicine and Animal Production, Naples, Italy (No. 0051420).

### 2.2. Experimental Protocol

All dogs underwent complete clinical and ophthalmic examination (visual examination, slit lamp biomicroscopy, STT-1 and intraocular pressure (IOP) evaluation) on day 0 according to the scheme reported in Table 2. For the STT-1, the STS (35-mm Whatman filter paper; Tiedra Farmacéutica S.L., Madrid, Spain) was applied to the lower conjunctival sac at the junction of the lateral and middle thirds (while avoiding touching the cornea) for 1 min with the eye closed, and the length of the moistened paper was recorded (for both eyes). Additionally, the IOP of both eyes was measured using a TonoVet^®^ rebound tonometer (Icare Finland Oy, Vantaa, Finland).

On day 1 we collected tears by STS and OS and performed the tear parameter analysis (Table 2).

The tear film BUT measurements and fluorescein and lissamine green staining were performed on the day after tear collection (day 2). For the BUT measurement, 10 μL of fluorescein solution was obtained by soaking a fluorescein impregnated paper strip in 1 mL of sterile normal saline for 1 min; the strip was placed in an empty syringe prior to drawing up the solution. Then, the solution was applied to the eye; the eyelids were closed, opened, and then held open until the tear film began to dissociate from the corneal surface with the formation of a central dry spot, while we were timing the process (in seconds). The eye was then irrigated with additional saline and the cornea was examined for dye retention in case of corneal ulcers or corneal erosions.

After the application of fluorescein, lissamine green was applied on each eye, to evaluate if this vital dye stained any dead and degenerating cells and mucous.

As reported in Table 2, all the procedures, including the clinical examination and the ocular parameter measurements, were performed in the following order to minimise the effect of the previous measurement. A 10-min interval between each test was established, and all tests were performed in the same order. All STS came from the same lot number to avoid any variation among them.

The same examiner performed all measurements between 09:00 and 12:00 a.m. All subjects were examined in the same laboratory, where the room temperature remained stable at 23 °C and the humidity at 40% (one room was selected and its temperature and humidity were checked every day during the study).

### 2.3. Complete Blood Count and Serum Biochemistry

To determine the health status of dogs, before ocular measurements and tear collection, haematobiochemical screening was performed on all dogs a week before all ophthalmic procedures. More specifically, 10 mL of blood was collected from each fasted dog enrolled in the study by jugular venipuncture. Each blood sample was divided into two aliquots to perform a complete blood cell count and serum biochemistry analysis. The first blood aliquot was collected in tubes containing potassium ethylene diamine tetra-acetic acid (EDTA) for a complete blood count (CBC), while the second blood aliquot was placed in tubes without an anticoagulant agent, allowed to clot, and centrifuged at 908× *g* for 15 min at 4 °C. The serum samples were stored at −80 °C and defrosted immediately before batch analysis.

CBCs were performed using a semi-automatic cell counter (Genius S; SEAC Radom Group, Calenzano, Italy).

A semi-automatic clinical chemistry analyser (OLOT; Spinreact, Girona, Spain) was used to analyse the azotemia, creatinine, glutamate pyruvate transferase (GPT), gamma glutamyl transferase (GGT), aspartate aminotransferase (AST) and total protein (TP) concentrations or activities (data not shown).

### 2.4. Tear Sample Collection

**Figure 1:**
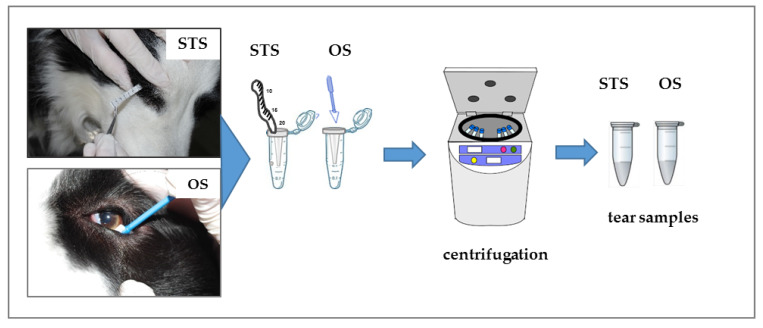
Phases of the experimental procedure for dog tear collection. STS: Schirmer tear strips, OS: ophthalmic sponges.

On day 1, tear samples were collected using a standardised STS (Schirmer-Plus^®^; GECIS, Neung-sur-Beuvron, France) or OS (Merocel^®^; Beaver-Visitec International, Inc., Waltham, MA, USA). In each dog, tear collection was performed by randomly using STS in one eye and OS in the other. The STS were inserted in the inferior conjunctival fornix of the eye. During sampling, the eyes were kept closed with slight finger pressure and after 60 s the strip was removed. After 15 min, tears were collected from the contralateral eye using an OS, which was kept in the lateral canthus and the conjunctival sac for 60 s (Figure 1). Care was taken to prevent damage to the conjunctival surface and local irritation. Tears were always collected by the same operator and without topical anaesthesia.

Following tear fluid sampling, the wetted STS and OS were transferred immediately to 0.5 mL tubes punctured at the bottom with a cannula. Each tube was placed in a larger (1.5 mL) tube and centrifuged at 16,000× *g* for 10 min at 4 °C. The centrifugal force pulled the tear fluid out of the STS and OS through the central pore at the bottom of the smaller tube and into the outer 1.5 mL tube [29] (Figure 1). The tear proteins were immediately collected and frozen at −80 °C until further analysis. The tear volumes absorbed (VA) and recovered in each sample were calculated based on the difference of the post- and pre-collection weights of the 0.5 mL and 1.5 mL tubes [30].

### 2.5. Tear Protein Analysis

Total proteins were determined by using the Bradford method [31] according to the manufacturer’s recommendation. The absorbance was measured at 595 nm by using a plate reader (Multiskan FC; Thermo Fisher Scientific, Waltham, MA, USA) as reported previously [32]. The protein concentrations were determined using a bovine serum albumin (BSA) calibration curve. More specifically, a standard microplate protocol was followed owing to the limited sample volume and considering that this method is more sensitive to proteins [33].

### 2.6. AQP1 Expression in Tears by Western Blotting

The tear samples were diluted in phosphate buffered saline (PBS) to a concentration of 0.5 μg/µL, and equal amounts of the total protein sample (20 μL) were loaded into wells. After adding NuPAGE™ LDS Sample Buffer (4×) (Invitrogen, Life Technologies, Carlsbad, CA, USA) and NuPAGE™ Sample Reducing Agent (10×), the prepared samples were heated at 95 °C for 5 min; then, the samples were loaded into 4–12% bis-acrylamide gel (SDS minigels; Invitrogen, Life Technologies, Carlsbad, CA, USA) filled with NuPAGE™ MES SDS Running Buffer (20×) and run at 160 V until the samples reached the bottom of the gel. Proteins were transferred on to nitrocellulose membranes using the iBlot Dry Blotting System (Invitrogen, Life Technologies, Carlsbad, CA, USA) according to a previously reported method [34,35]. Briefly, the membrane was incubated first with blocking solution (5% milk in tris-buffered saline with Tween 20 (TBST)) and then with primary rabbit polyclonal AQP1 antibody (ab–15080, 1:500; Abcam, Burlingame, CA, USA) overnight at 4 °C. Thereafter, the membrane was washed three times and incubated with secondary goat anti-rabbit antibody conjugated with peroxidase (111-005-003, 1:4000; Jackson ImmunoResearch Laboratories, Inc., West Grove, PA, USA) at room temperature for 1 h. Finally, the membrane was incubated with WesternBright Sirius, a femtogram ECL HRP substrate (Advansta, San Jose, CA, USA) and scanned with a C-Digit Blot Scanner (LI-COR Biotechnology Lincoln, NE, USA).

To monitor the loading of gel lanes, the same blots were stripped and reprobed using an anti-actin monoclonal antibody (A4700, 1:1000; Sigma Chemical Co., St. Louis, MO, USA) as the primary antibody and secondary goat anti-mouse antibody conjugated with peroxidase (111-035-003, 1:5000; Jackson ImmunoResearch Laboratories, Inc., West Grove, PA, USA). The intensity of protein bands was quantified by densitometry analysis using NIH Image J software (NIH, National Institute of Health, Bethesda, MD, USA). In particular, the relative signals from each sample were determined by comparing the intensity of the AQP1 band with that of β-actin, considering that the same blot membrane was stripped and reprobed against β-actin.

### 2.7. Statistical Analysis 

All the ocular parameter data were presented as mean ± standard deviation (SD). Student’s t-tests were used for the analysis of the tear volume (µL) and protein concentration (µg/µL) of the samples collected either by STS or by OS; these data were also presented as median ±SD. Additionally, the coefficient of variation (CV = standard deviation/mean) was calculated for these data according to Keech et al. [36]. Moreover, considering the importance of tear fluid dynamics for the maintenance of ocular surface health, Spearman’s correlation coefficients were calculated to determine the correlations among the different tear parameters examined. The correlation was described as weak, moderate, strong and very strong when the correlation coefficient (r) was 0.000–0.250, 0.250–0.500, 0.500–0.750 and 0.750–1.000, respectively. *p* values less than 0.05 were considered statistically significant.

## 3. Results

### 3.1. Ocular Parameters

A summary of the ocular parameters examined (STT-1, IOP and BUT) is shown in Table 3. 

**Figure 2:**
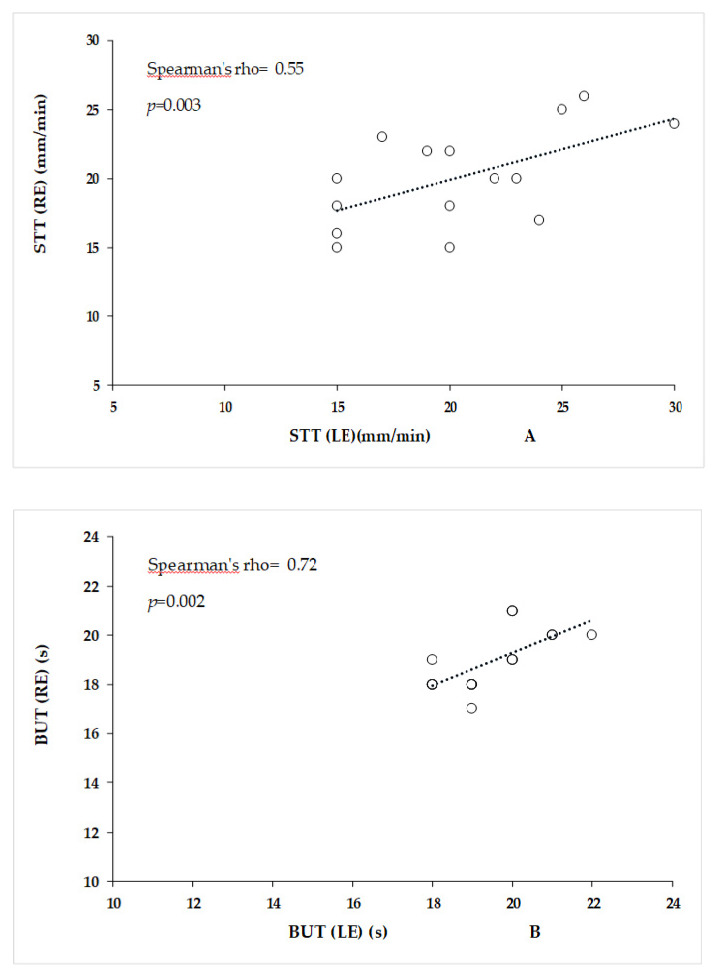
Shirmer tear test (STT-1) (**A**) and break up time (BUT) (**B**) correlations between the right (RE) and left eyes (LE).

The fluorescein and lissamine green stains produced negative results in all the tested dogs. The STT-1 (≥12 mm/min), IOP (<25 mm Hg), and BUT (>10 s) values where within the physiological ranges in all dogs examined. These values demonstrated a normal production of the aqueous components in the tear film, normal aqueous humour dynamic processes, and a normal rate of tear evaporation (indirectly indicating normal mucous and lipid components in the preocular film) in dogs included in this study. In addition, the statistical analysis using Student’s *t*-test showed no significant differences in STT-1 (*p* = 0.41), IOP (*p* = 0.35) and BUT (*p* = 0.12) between the right and left eyes, indicating no influence on the eyes during ocular measurements (Table 3). In addition, a comparison between the left and right eyes revealed a significant correlation (Spearman rho, strong correlation) between STT-1 and BUT (Figure 2).

The correlation between the STT-1 and BUT values in the right and left eyes indicate the uniformity in tear film characteristics between the eyes of the same subject in healthy dogs. These findings confirm that each eye should be considered separately for further evaluation of tear protein expression, using the two adopted collection methods (STS and OS).

### 3.2. Analysis of Dog Tear Parameters (Volume and Total Protein Content)

**Figure 3:**
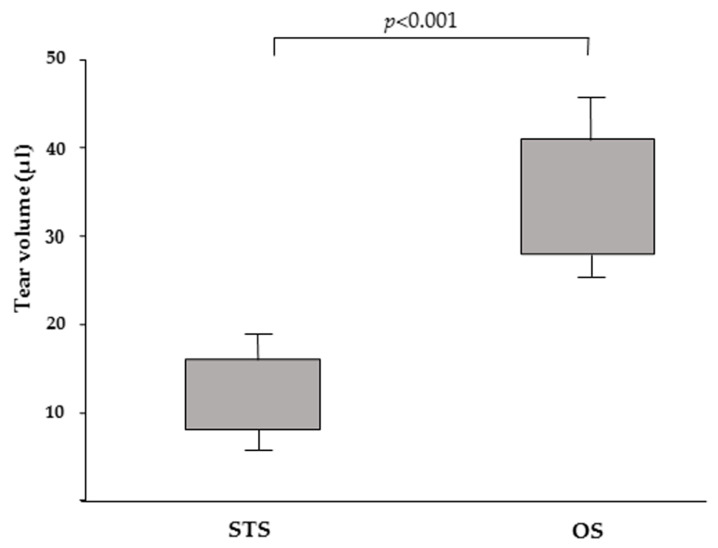
Box and whisker plots depicting the volume of tears collected by STS and OS.

The median tear volume by STS and OS collection was 12.1 µL (range 8–18 µL) and 35.5 µL (range 28–45 µL), respectively, a difference that was statistically significant (*p* < 0.001, Figure 3). The CV calculated for STS and OS was 26.8% and 18.5%, respectively. These indicate a different capacity of the STS and OS matrix to release tear fluid.

**Figure 4:**
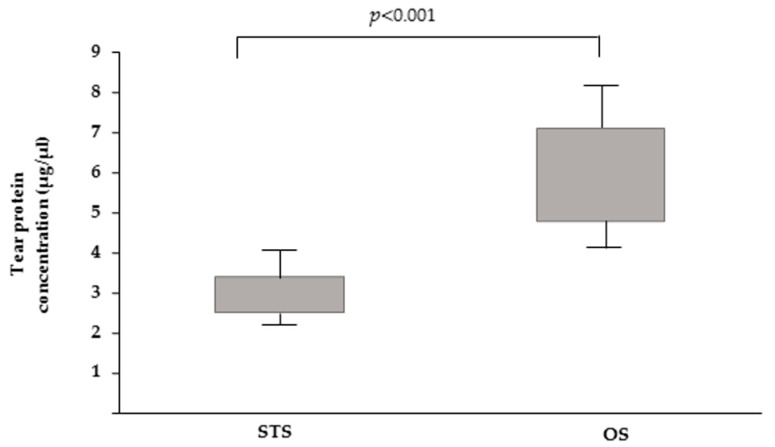
Box and whisker plots depicting the total protein concentration of tears collected by STS and OS.

The tear protein concentration determined using the STS and OS methods was 3.3 ± 0.6 (µg/µL) and 6.22 ± 1.11 (µg/µL), respectively (Figure 4). The statistical analysis performed using Student’s *t*-test showed a significant difference (*p* < 0.001). In addition, the CV calculated for the STS and OS was 20.3% and 17.8%, respectively. These results demonstrated that ophthalmic sponge is efficient in quickly absorbing tear fluid and retaining it in the pores (material density 1.1/cm^3^, absorption speed <10 s, and liquid retention volume 12×) as described in the datasheet using Merocel^®^ (Beaver-Visitec International, Inc., Waltham, MA, USA).

**Figure 5:**
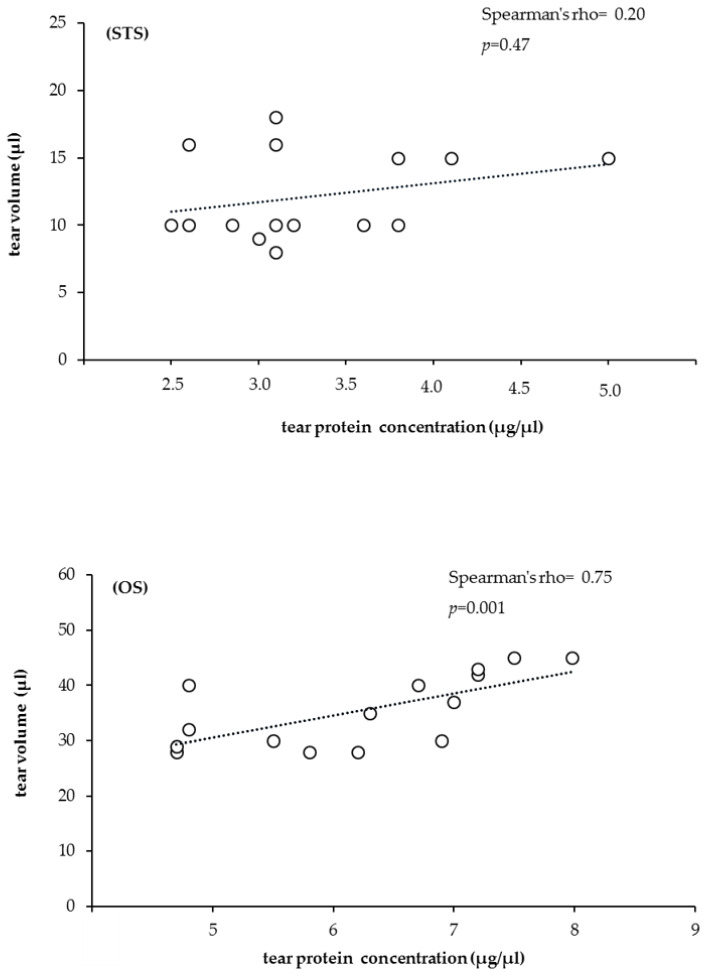
Spearman’s rank correlations testing for tear volume (µL) and concentration (µg/µL) of tear samples collected by STS (**A**) and OS (**B**).

Moreover, the results of Spearman’s correlation showed a moderate correlation between tear volume and total protein concentration in the STS tear collection method (Spearman’s rho = 0.20). Contrarily, a strong correlation was observed between these parameters (Spearman’s rho = 0.75) in the OS collection method (Figure 5). This elevated protein concentration (observed empirically by the turbidity of the extracted tear fluid) could be indicative of negligible protein loss using the method with Schirmer strip.

### 3.3. AQP1 Expression by Western Blotting 

**Figure 6:**
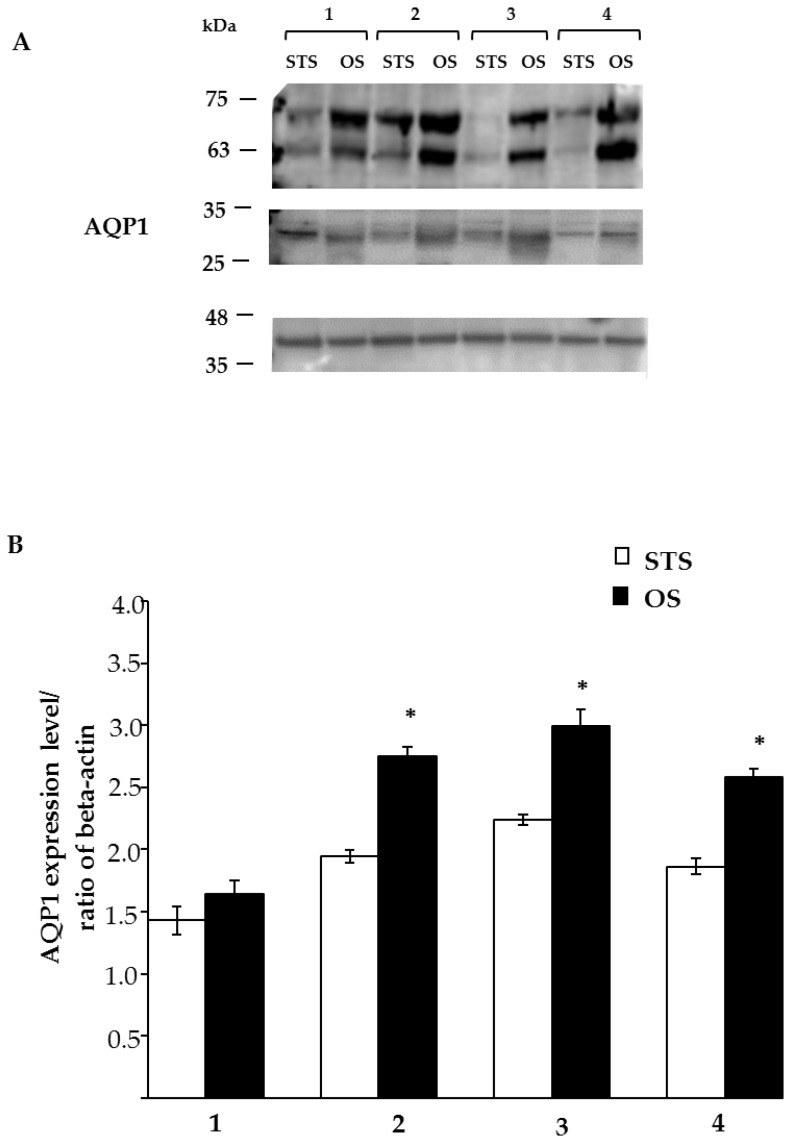
Representative Western blot of AQP1 protein isolated from tears collected by the STS and OS methods. (**A**) Image is representative of three distinct experiments performed on four dogs (lanes 1–4). The expression of AQP1 was normalised against that of β–actin. (**B**) Densitometric analysis of AQP1 expression.

The results of Western blotting analysis showed the presence of AQP1 in tears by a specific band corresponding to the protein with a molecular weight of approximately 28 kDa (Figure 6A). More specifically, the results showed a difference in the tears collected with the two methods, with more intense bands for the protein in tears collected by the OS method. This difference was evidenced further by densitometry (Figure 6B), in which we calculated the AQP1 to beta actin expression ratio, with the latter used as a protein loading control. The difference determined by Student’s t-test was statistically significant (*p* < 0.001). 

## 4. Discussion

In this study, we demonstrated for the first time the expression of AQP1 in dog tears by Western blot analysis. Our results demonstrate the presence of AQP1 in tears, indicating a potential role of this water channel protein in eye homeostasis, relative to other ocular AQPs whose specific functions have been elucidated [12,14,37]. 

Among different AQPs distributed in the eye, AQP1, AQP3, AQP4 and AQP5 appear to be more extensively expressed in the lacrimal gland as observed in humans [17] and laboratory animals [5,17,38]. The role of these channel proteins in relation to the lacrimal gland requires a careful analysis considering the complex organisation of the ocular structure.

The lacrimal gland together with the ocular surface (cornea, conjunctiva and Meibomian gland) and the interconnecting sensory and motor innervations constitute an integrated system called the lacrimal functional unit (LFU). This system along with different components contributes to the homeostatic microenvironment regulation. Studies have showed that AQP1 is directly or indirectly involved in the eye functions.

**Figure 7:**
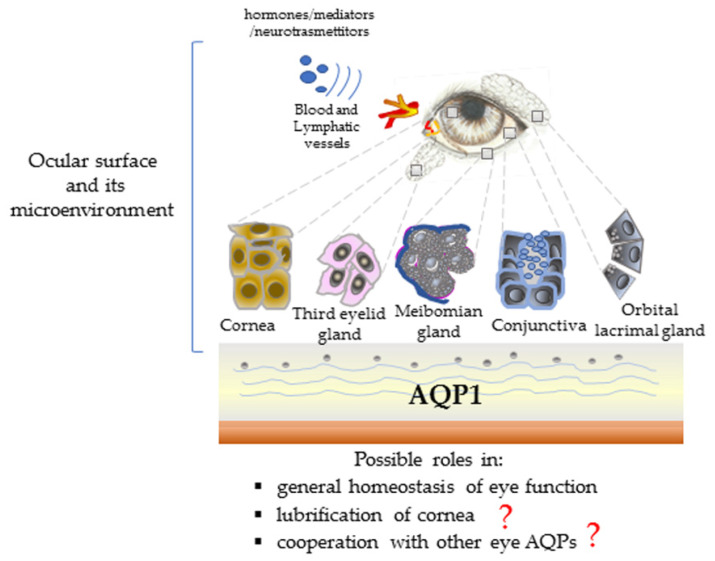
Scheme illustrating the possible roles of AQP1 in the lacrimal functional unit (LFU) of the dog.

AQP1 has been described in the microvascular endothelia of the lacrimal gland [5,13,38,39] and in nasolacrimal duct epithelium, suggesting that it can support the absorption and/or secretion of tear components [40]. Moreover, although AQP1 null mice did not show clinical signs of keratoconjunctivitis sicca (KCS), only tear fluid volume and chloride concentration were evaluated in these experimental conditions, while a specific evaluation of tear fluid composition has not been conducted [5]. In addition, some functional studies have suggested that both AQP1 and AQP5 in the cornea act as major components of an osmotically driven water movement pathway across the corneal epithelium and endothelium, respectively [41]. In dogs, the expression and localisation of AQPs have not been investigated in nasolacrimal ducts or lacrimal glands that produce the aqueous component of canine tears—the lacrimal gland of the orbit and the lacrimal gland of the nictitating membrane. It can be hypothesised that AQP1 contributes to different functions of lacrimal fluid that is distributed throughout the surface of the eye, interacting with the conjunctiva and cornea. These findings suggest the role of AQP1 in controlling the homeostasis of the tear fluid and in its interaction with ocular surface contributing (probably in association with other AQPs) to the ocular microenvironment (Figure 7).

Alternatively, it is possible that AQP1 ends up in dog’s tears by leakage. However, it cannot be determined from which anatomical structure it comes from; for example, from the lacrimal glands of the orbit and nictitating membrane, or from the corneal stroma during transendothelial osmotic water transport from aqueous to stroma accompanied by osmotic transport from stroma to tears [42]. Recently, the results of a study on rabbit AQP mRNA expression suggested the presence of AQP1 in the conjunctiva in this species, although protein expression could not be detected by immunohistology [43]. Further studies should be performed on AQP1 expression in the anterior compartment of canine eye under physiological and pathological conditions.

In addition, the possibility that tears may be a vehicle for AQP1 delivery across a damaged corneal surface, following a scratch wound or surgical intervention with partial-thickness corneal stromal debridement, provides novel therapeutic possibilities, considering that AQP1 is involved in keratocyte migration [44] and corneal transparency [42] in the corneal healing processes.

The expression of AQP1 in dog tears is related to the tear collection method. Our results showed a higher AQP1 expression in the tears collected using the OS method relative to the STS method suggesting the suitability of this method to collect tears. In addition, our results showed a high protein concentration and a strong correlation between the tear volume and total protein concentration using the OS method (Spearman’s rho = 0.75, *p* = 0.001) These date are in agreement with the results of Lee et al. [45] obtained using equal Merocel^®^ sponge for tear collection. Similarly, the results of a study, which aimed to compare several OS and extraction buffers to optimise a tear collection method for cytokine quantification using the luminex technology, demonstrated that many cytokines were recovered from Merocel^®^ sponges more efficiently than from other sponges. This result was attributed to the specific chemical composition of the sponges. In particular, the Merocel sponge has 100% open pores with no dead-end pockets that hold residues [46]. In addition, cellulose sponges are six times more absorbent than filter papers [47], which may reduce the effects of evaporation [48]. However, albeit during the past few years, several tear collection methods (such as microcapillary glass tubes, STS and OS) have been developed, and their applicability and efficacy have been assessed [46,49], numerous questions remain unanswered. The findings of the present study demonstrate the efficiency of the sample processing methods.

Based on these considerations, the results of our study could aid in developing further tests and biochemistry studies to characterise tear fluid in terms of ocular health and disorders.

However, the following study limitations have been noted. The methods of collecting dog tear samples as well as the number of dogs must be increased. For example, Spearman correlation analysis revealed a good relationship between tear volume (STS and OS collection method) and age of dogs, while, as expected by other studies, no correlation was observed with respect to dog body weight (data not shown). In addition, any possible gender difference needs to be examined, as our study had a high percentage of male dogs than female dogs. Our findings can be supported by other analysis to demonstrate, using a larger population size, the reproducibility of tear collection methods as well as the specific mechanisms (osmotic pressure and electrostatic forces) involved in the efficacy of the OS collection method. This additional information could be useful to adapt tear collection protocol to assess the correlations observed in tear diseases (i.e., dry eye).

## 5. Conclusions

The Western blot analysis of dog tears identified AQP1 as a band of 28 kDa. The results showed a difference in the tears collected with the two collection methods, with a greater band intensity in the protein in tears collected by the OS method.

Although we recognise that the number of dogs enrolled in the study is limited, our data shed light on the role of AQPs in tear fluid production and homeostasis, hypothesising their involvement in several eye diseases. Such studies will allow the development of new diagnostic tests and innovative therapies for ophthalmic diseases (related to changes in hydration status and to alterations of tear fluid composition) based on AQP expression.

## Figures and Tables

**Table 1:** Characteristics of the 15 dogs enrolled in the study.

ID	Breed	BW (kg)	Sex	Age (years)
1	Labrador	32	F	2
2	Golden Retriever	34	M	5
3	Kurzhaar	28	M	3
4	Mix breed	10	F	2
5	Whippet	17	M	6
6	Whippet	19	M	7
7	Mix breed	30	M	3
8	Mix breed	31	M	4
9	Mix breed	25	F	5
10	Jack Russel	13	M	3
11	Beagle	17	M	6
12	Mix breed	19	M	1
13	Mix breed	14	F	3
14	Border Collie	15	F	2
15	Pointer	23	M	1

**Table 2:** Study procedures and order of testing.

Order of Testing	Study Procedure
	**Day 0**
1	Visual examination
2	Slit lamp examination of eyelids, cornea, tear meniscus and conjunctiva
3	Shirmer tear test (STT-1) and intraocular pressure (IOP) (both eyes)
	**Day 1**
1	Tear sample collection: by randomly using Schirmer tear strips (STS) in one eye and ophthalmic sponges (OS) in the other
2	Analysis of parameters (volume and total protein content)
3	Evaluation of aquaporin 1 (AQP1) expression by Western blotting
	**Day 2**
1	Assessment of tear film break up time (BUT)
2	Fluorescein test corneal staining
3	Lyssamine green corneal staining

**Table 3:** Summary of ocular parameters examined.

ID	STT-1 (RE) (mm/min)	STT-1(LE) (mm/min)	IOP (RE) (mm/Hg)	IOP (LE) (mm/Hg)	BUT (RE) (s)	BUT (LE) (s)
1	15	18	15	20	18	19
2	20	22	16	22	19	17
3	22	20	21	14	18	18
4	15	15	14	17	21	20
5	30	24	22	19	20	21
6	13	16	14	20	19	18
7	17	24	16	22	18	19
8	18	20	23	21	20	22
9	25	25	22	23	18	18
10	23	17	20	22	18	19
11	23	20	17	17	20	19
12	26	26	19	21	21	20
13	20	15	23	19	20	19
14	15	20	14	18	20	21
15	19	22	14	17	18	18

Schirmer tear test (STT-1), intraocular pressure (IOP), break up time (BUT), right eye (RE), left eye (LE).
